# Increased Employment for Segregated Roma May Improve Their Health: Outcomes of a Public–Private Partnership Project

**DOI:** 10.3390/ijerph16162889

**Published:** 2019-08-13

**Authors:** Lucia Bosakova, Andrea Madarasova Geckova, Jitse P. van Dijk, Sijmen A. Reijneveld

**Affiliations:** 1 Department of Health Psychology, Medical Faculty, P.J. Safarik University in Kosice, Trieda SNP 1, 040 11 Kosice, Slovakia; 2 Graduate School Kosice Institute for Society and Health, P.J. Safarik University in Kosice, Trieda SNP 1, 040 11 Kosice, Slovakia; 3 Olomouc University Society and Health Institute, Palacky University in Olomouc, Univerzitni 22, 771 11 Olomouc, Czech Republic; 4 Department of Community and Occupational Medicine, University Medical Center Groningen, University of Groningen, Antonius Deusinglaan 1, 9713 AV Groningen, The Netherlands

**Keywords:** deprivation, Roma health, health promotion, unemployment, employability

## Abstract

Increasing employment opportunities for segregated Roma might prevent major economic losses and improve their health. Involvement of the private sector in Roma employment, on top of intensified governmental actions, is likely to be a key to sustainable improvement, but evidence on this is scarce. Our aim was to determine the potential outcomes of such a partnership regarding increased employability and the resulting improved well-being and health. We therefore investigated a Roma employment project called Equality of Opportunity, run since 2002 by a private company, U.S. Steel Kosice, in eastern Slovakia. We conducted a multi-perspective qualitative study to obtain the perspectives of key stakeholders on the outcomes of this project. We found that they expected the employability of segregated Roma to increase in particular via improvements in their work ethic and working habits, education, skills acquisition, self-confidence, courage and social inclusion. They further expected as the main health effects of increased employability an improvement in Roma well-being and health via a stable income, better housing, crime reduction, improved hygienic standards, access to prevention and improved mental resilience. Social policies regarding segregated Roma could thus be best directed at increasing employment and at these topics in particular to increase their effects on Roma health.

## 1. Introduction

Roma unemployment in Central and Eastern Europe (CEE) is very high; e.g., it is estimated to be 71.0% in Slovakia, 41.5% in Bulgaria, 40.5% in the Czech Republic, 52.5% in Hungary and 35.5% in Romania [1], and this likely affects Roma health negatively. In Slovakia, Roma represent one of the largest ethnic minority groups, with a substantial portion of so-called segregated Roma or the Roma underclass. This latter underclass has high unemployment rates (close to 100% in some rural areas), with mostly permanent unemployment, low education attainment levels and qualifications, a lower living standard, bad housing conditions and a strong dependency on social support [2,3,4]. Stereotypes, discrimination, poor education and often almost no work experience have led to poor access to the labour market and an increased distance from the labour market of this population [3,5].

Poor access to employment, together with low education and bad housing, contributes to a range of avoidable poor health outcomes for this community [6,7]. Examples include a high prevalence of chronic disease, poor dental health and difficulties in seeing and hearing properly, among others [8]. To improve health outcomes of Roma, reducing unemployment should be a priority [2]. Improving employment has been shown to improve health, though not in all cases [9]. Goodman [9] suggests that the creation of employment alone does not guarantee a positive impact on health, while Stateva et al. [10] emphasise that a more comprehensive approach regarding increased employability is needed.

A public–private partnership might help to increase the employability of segregated Roma. Public and private partners have been shown to separately not have sufficient capital to create a sustainable solution for the employability of segregated Roma [2]. However, combining their assets could make successfully influencing employment much more likely via offering special work opportunities. Pivotal for this success is to create appropriate opportunities for segregated Roma, who only with difficulty are able to use their current potential to break their vicious circle of poverty. Besides intensified public employment policies regarding this group, cooperation with the private sector might be a key to increasing Roma employment [11,12]. The major considerations behind such a partnership might be a greater variety of job opportunities for low educational levels and the financial sustainability of the offer.

Unfortunately, evidence on the potential and actual effects of public–private partnerships for increasing Roma employment is scarce and is completely lacking regarding improving Roma health via increased employability. The available evidence on increasing Roma employment mainly regards municipal enterprises [3,13], social enterprises [14], governmental and non-governmental programs [15] and private Roma employment projects [2], and has a rather descriptive character. Such separate approaches often have many weaknesses; we suggest that combined public–private approaches are needed.

Multi-perspective comprehensive studies on public–private partnerships aiming to increase Roma employability and thus this improve Roma health are lacking. This paper therefore aims to determine the potential outcomes of a public–private Roma employment project regarding increased employability and resulting improved well-being and health.

## 2. Materials and Methods

### 2.1. Design

We conducted a multi-perspective qualitative study investigating a Roma employment project called Equality of Opportunity, established in 2002 by U.S. Steel Kosice (USS Kosice). This project in terms of size, duration (ongoing), complexity and sustainability represents an interesting example of an attempt to address the Roma social inequality issue. We used the Context, Mechanism, Outcome (CMO) framework to structure data collection. The CMO configuration makes up part of the realist evaluation approach [16] and intends to yield a proposition stating what works for whom and in what circumstances. This should increase the understanding of the effectiveness of the programme, with an explanation of why the outcomes developed as they did, how the project was able to act on the underlying mechanisms, and in what contexts [17]. We will report only on outcomes in this paper.

The study consisted of the following phases. First, we established a protocol. Next, we collected data on the setting of the project (project background). Third, we collected data using direct observation, in-depth semi-structured interviews, focus groups and informal face-to-face unstructured interviews. Last, we analysed data in two rounds: the first round ran along with the data collection, and the second round was performed later. More detailed information on these stages can be found in Appendix A.

### 2.2. Sample

We included the main actors in the project, i.e., the Roma community, professionals (labour, education), public authorities and others (a priest, a nun, a cultural anthropologist) with proper methods of data collection for each group. The final sample for both the formal and informal interviews consisted of 20 respondents (55% male), for the focus groups 28 respondents (39% male), and for direct observation 98 respondents (gender not monitored) (Table 1).

### 2.3. Procedure

We collected data using in-depth semi-structured interviews, unstructured interviews, focus groups and direct observation. The in-depth semi-structured interviews were carried out with the aim of systematically covering all topics of interest (outcomes, with a special focus on well-being and health and health equity). These interviews were performed face-to-face, collecting the data by audio recording with informed consent from participants and by written field-notes. The layout and structure of the interviews are presented in Appendix B.

Informal unstructured interviews were carried out to gain the views of people not directly involved in but possibly affected by the project. These interviews were done by one researcher accompanied by a Roma community worker who, in case of need, also translated from and to the Romani language. The researcher collected data using written field-notes.

Focus groups were performed to gain the views of people not directly involved in but possibly affected by the project. We performed three focus groups, each by three researchers, with the contents of interviews collected by written notes. The focus group with Roma children was facilitated by a Roma assistant who, in case of need, also translated from and to the Romani language. The layout and structure of the focus group scenario are presented in Appendix C.

Direct observation was used during the recruitment process to examine participants, their settings and their practices when applying for a job in the project. During this recruitment, we also observed attitudes and habits of USS Kosice representatives. Furthermore, the residences of participants were visited directly in an effort to capture life in the settlements. During the visits to the settlement, researchers were without USS Kosice representatives, accompanied only by local community workers who, if needed, also translated from and to the Romani language.

### 2.4. Measures

We collected data on the setting of the project and on the expected effect on employability and improved well-being and health. Regarding the project setting, we collected data on the launch of the project, including its circumstances and key persons, the project’s main goal, type of contracts, financial remuneration and characteristics of the project participants. Regarding the characteristics of the project participants, we examined gender, locality, age and education. Age structure, educational level and average number of workers were calculated based on the overall number of participants since the beginning of the project in 2002. Regarding employability and well-being and health, we further collected data using the CMO framework. This regards only the outcome measures of the CMO framework. Examples of the questions regarding outcomes of the CMO framework are listed in Table 2.

### 2.5. Analysis and Reporting

First, we described the setting of the project (project background). Second, we assessed the factors potentially leading to increased employability of segregated Roma using the CMO framework. Third, we assessed the resulting well-being and health, again using the CMO framework. For the second and third steps, we performed a content analysis of the data based on recurrent abstraction, i.e., repetition of reading and summarising in steps of data coded as relating to the same topics or variables [18]. The content analysis was performed separately by three researchers with the aim of finding common themes (searching for themes, reviewing themes, defining and naming themes) in order to identify contexts, mechanisms and outcomes as seen by the stakeholders. Both written notes and the recordings, after transcription, were coded manually. The answers regarding the outcomes were given sub-codes, as in a typical qualitative analysis. Next, separate results given by three researchers were then compared, while differences of opinion were discussed and resolved. Lastly, the final version was discussed, agreed and finalised.

## 3. Results

### 3.1. Project Setting

The Equality of Opportunity project is fully financed by USS Kosice, the largest private employer in the region of eastern Slovakia, in cooperation with the municipalities comprising the adjacent Roma settlements. USS Kosice offers Roma jobs with a significantly higher salary than the minimum wage and with training, while municipalities cover the selection of the candidates. Successful candidates formally become employees of the municipality, but are assigned to USS Kosice for temporary work. USS Kosice has created around 170 jobs for segregated Roma since 2002. More extensive information can be found in Bosakova (2018) [19].

Project participants were all males and came mainly from the three adjacent settlements, which were within a 15 km radius of the USS Kosice plant. Ages of participants ranged from 18 to 60 years, and nearly three-quarters were aged 21–40 years. Most project participants had completed only primary school (Table 3).

### 3.2. Increased Employability of Segregated Roma

**Figure 1:**
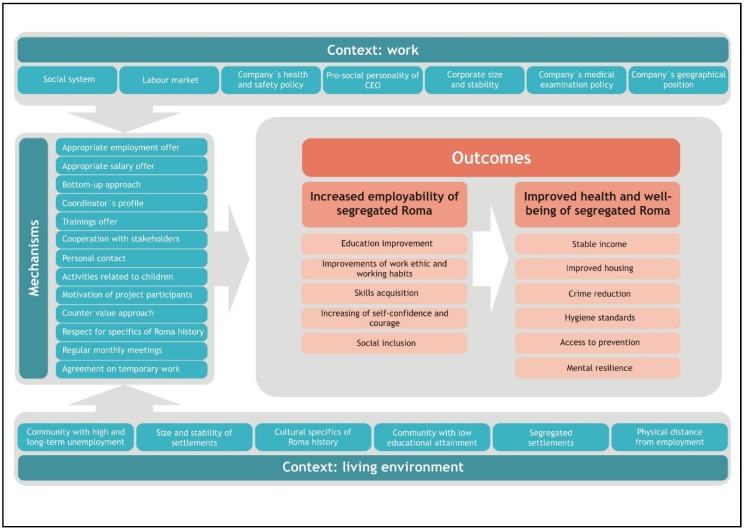
CMO framework of factors leading to increased employability of segregated Roma leading to their better well-being and health.

Informants perceived the following outcomes to be related to increased employability: improvement of work ethic and working habits, education improvement, and skills acquisition. Furthermore, an increase of self-confidence and courage and of social inclusion (Figure 1) were mentioned. All these factors were mentioned by all types of informants. For illustrative quotes related to the above section of Results, see Table 4.

### 3.3. Resulting Better Well-Being and Health of Segregated Roma

Regarding better well-being and health in segregated Roma as a result of increased employability, informants mentioned several outcomes in which they perceived improvement. These regarded: a more stable income through the job, improvement of precarious housing, and crime reduction. Furthermore, they mentioned several factors that in their perception had improved the health of the participants: an improvement of hygienic standards, an improvement of access to prevention, and an improvement of mental resilience (Figure 1). All factors were mentioned by all types of informants. For illustrative quotes related to the above section of Results, see Table 4.

Table 4 provides some examples of the narratives of the different interviewed agents and how they were obtained (e.g., focus groups, interviews, etc.) for the topics that frequently arose during the data collection.

## 4. Discussion

We explored the potential outcomes of a public–private Roma employment project in terms of employability, and the resulting improved well-being and health, based on key stakeholders’ perspectives. We found that they thought Roma employability might increase via an improvement of their work ethic and working habits, education, skills acquisition and an increase in self-confidence and social inclusion. We also found several areas of well-being and health of segregated Roma that could improve via their increased employability, the most important being a stable income, an improvement of precarious housing, crime reduction, better hygienic standards, improved access to prevention and better mental resilience.

### 4.1. Outcomes Related to Increased Employability of Segregated Roma

We found that informants considered Roma employability to have increased due to improvement of in their work ethic and working habits (e.g., consistently good performance and attendance levels). This is crucial, taking into account the situation of segregated Roma as having typically lost working habits and having limited working experience, which affects their access to the job opportunities [3]. We did not find any evidence that would confirm or disprove our informants’ expectations that improved working habits increase the employability of segregated Roma. Active labour market programmes (ALMPs) are themselves based on preserving good working habits by integrating the unemployed into work rather than providing passive income support [20], so a positive impact is expected. However, services such as monitoring and counselling post-employment are almost non-existent, which might lead to critical gaps in Roma workforce development [21]. The monitoring of the work trajectories of segregated Roma and other hard-to-employ groups seem to be important in order to determine whether improved working habits really do increase employability in a way that is sustainable on the labour market.

Improvement of education was seen by informants as another crucial outcome that increases employability and through this improves health. The relationship between education and unemployment has been extensively substantiated [21,22,23], as has that between education and health [24,25]. Roma are significantly less educated than non-Roma [26], and the employment gap of Roma has been shown to be strongly related to their low education [21,26]. Reducing the education gap is important to prevent unemployment and the reproducing of poverty in future generations. Public–private partnerships might help here at least in regard to vocational training.

Skills acquisition, i.e., obtaining certificates and also social skills, were key topics in increasing employability according to informants. Our finding is in line with other studies pointing out the importance of skills development in order to be successful in the labour market [3,13,27]. Sufficient opportunities for segregated Roma to develop their skills are key in order to increase their employability and might be ensured by public–private partnerships.

Furthermore, informants reported more self-confidence, self-esteem and courage as another outcome that increases employability, which also aligns with other research [9,13]. Self-confidence, self-esteem and courage might project into self-efficacy, a related concept more focused on the belief in one’s own abilities to meet the challenges ahead and to succeed [28], which is crucial in relation to employability. However, self-confidence, self-esteem and courage are substantially lacking in segregated Roma, similarly to other excluded communities, with causes and effects probably being related. This can represent a serious barrier to employability and should be addressed separately by social policies. Finally, social inclusion was reported by informants to be an important outcome related to the employability of segregated Roma. This aligns with previous studies declaring that employment plays an important role in fostering integration in societies, as it functions not only as a source of monetary income, but also as a tool of social inclusion [29]. Stateva et al. [10] also suggest that social inclusion and employment are interconnected, and social inclusion happens mainly through employment. Occupational integration of segregated Roma seems to be key predictor of their overall integration.

However, an increased employability of segregated Roma will not always increase their employment opportunities because other factors also affect the degree to which they get a job. One important factor is anti-Gypsyism, i.e., specific racism towards Roma that regards a wide spectrum of discriminatory expressions and practices including many implicit or hidden manifestations [30]. Anti-Gypsyism provides a structural mechanism that reduces the employment of Roma via effects on employers, potential colleagues and Roma themselves [6,31]. This mechanism should be seriously considered and tackled, as it may block the positive effects of increased Roma-employability. The current project was initiated by an employer, i.e., USS Kosice, showing that anti-Gypsyism from the side of the employer had been counteracted at least at a considerable degree. The increase of self-confidence on the side of the Roma can be interpreted as a sign of decreased internalization of anti-Gypsyism on the part of Roma themselves. Evidently, this issue deserves attention in other settings as well.

### 4.2. Better Well-Being and Health of Segregated Roma Resulting from Increased Employability

Informants expected better well-being and health to result from increased employability; in particular, a more stable income, improvement of precarious housing, crime reduction, improvement of hygienic standards, improvement of access to prevention, and improvement of mental resilience. All of these factors have previously been shown to have a positive impact on well-being and health [32,33,34,35,36].

A more stable income, improved housing, improved hygiene and crime reduction as important elements of the physical environment were considered by informants as outcomes of increased employability to have an impact on well-being and health. Regarding income, a higher and stable income in general enables the purchase of healthier food and/or investment in better health care, housing, schooling and recreation [9,36], and it prevents psychosocial stress. Regarding housing, our findings align with previous reports of Belak et al. [6], that the majority of the houses in segregated Roma settlements are built illegally and of poor quality. They are typically overheated with damp air and cold walls, overcrowded, often without access to water and a sewage system, and only illegally connected to electricity. Therein, a move from shanty housing to public-assistance dwellings or even one’s own legal house undeniably results in better well-being and health. Improved housing is closely connected to the improvement of hygienic standards mentioned by informants as well, enabled by access to water, to a sewage system, to electricity and to heating, which rapidly decreases the presence of parasites (e.g., lice, fleas) and frequent intestinal infections; contamination of public space by urine, faeces and smoke, and the presence of rodents; constant ergonomic strain, etc. [6]. With regard to crime reduction, we assume this might support the broken windows theory [37,38], explaining that visible signs of crime, anti-social behaviour and civil disorders create an urban environment that encourages further crime and disorder, including serious crimes.

Informants also mentioned improved access to prevention and improved mental resilience as other outcomes of increased employability related to well-being and health. With regard to prevention, Belak et al. [6] explain that segregated Roma often consider prevention to be unnecessary; thus, any improvement of access to prevention via increasing preventive medical examinations and increasing vaccinations is seen as improving health. Regarding mental resilience, we presume that our results are in accordance with a study suggesting improved mental health for unemployed who find new jobs [39] and the theory that work plays a central role of people’s lives [40]. Paid work provides both manifest benefits (associated with income) and latent benefits (associated with meeting psychological needs) [41,42]. People primarily engage in paid work to attain manifest benefits, but while employed, they also profit from its latent benefits [41,42].

If Roma employment increases due to their improved employability, this may not automatically increase their well-being and health as some jobs are simply not appropriate to reach that improvement. In many cases, Roma only have access to precarious jobs with low wages [5], which may not result in better well-being and health. This may also be another representation of anti-Gypsyism as discussed. Public–private partnership has; however, a huge potential to tackle anti-Gypsyism not only by increasing assets in terms of offering equal work opportunities, but also in terms of initiating the much needed dialogue within the business community regarding zero tolerance to discrimination and prejudice in the workplace [2]. These issues should be addressed in order to indeed realise a better well-being and health of segregated Roma as result from their increased employability.

### 4.3. Strengths and Limitations

Our study has several strengths, the most important ones being its wide range of informants and sources, which enabled various perspectives to be identified. This approach increased the robustness and transferability of the findings. However, some limitations need to be mentioned. We used a qualitative design, which does not allow a full quantitative generalisation of findings. Furthermore, social desirability may have affected responses. However, we used a multi-informant strategy to get a full picture, which resulted in no major problem of information depth and reduced the impact of this potential bias.

### 4.4. Implications for Practice and Policy, and for Research

The results of our study have implications for improving social policies. They imply that policy-makers should predominantly focus on the occupational integration of segregated Roma, which seems to be a key predictor of their overall integration, resulting in better well-being and health. This implies better monitoring and coaching of the work trajectories of segregated Roma, i.e., not only prior to entering the labour market but also during employment. Furthermore, social policies should focus on reducing the education gap and should also create sufficient opportunities for segregated Roma to develop their skills in order to prevent the re-deepening of unemployment and reproducing of poverty in future generations. Also, the lack of self-confidence, self-esteem and courage among segregated Roma, which represent serious barriers to employability, should be address by social policies. Policy-makers should consider the above-mentioned outcomes and health equity to be the ultimate outcome of decreasing unemployment and successful social policies, thus fully covering the up-stream causes of health inequalities. However, to reach this ultimate goal, continuous community health work, such as health mediation, including further guidance regarding the use of additional income, prevention, health literacy, etc., should be involved. Regarding research, a next step is definitely to examine the potential mechanisms of a public–private Roma employment project, including the appropriateness of the employment offered [5], as well as anti-Gypsyism [43], which definitely deserves separate attention. Future research should also aim to assess the effects of interventions on the employability of Roma on their long-term well-being and health, which may yield large gains in the health of this deprived group.

However, self-confidence, self-esteem and courage are substantially lacking in segregated Roma, similarly to other excluded communities, with causes and effects probably being related. This could represent a serious barrier to employability, and should be addressed separately by social policies.

## 5. Conclusions

We conclude that within the context of the segregated Roma community characterised by high and long-term unemployment and low education, an appropriate employment project based on public–private partnership might increase employability, particularly through improvement of their work ethic and working habits, education, skills acquisition, increased self-confidence and courage and social inclusion. Furthermore, increased employability also improves their well-being and health via a stable income, better housing, crime reduction, increased hygienic standards, a better approach to prevention and improved mental resilience. This study shows that public–private partnerships focused on providing special work opportunities to Roma from segregated settlements may largely contribute to their well-being and health.

## Figures and Tables

**Table 1:** Characteristics of the samples participating in the various data collection methods.

	Roma Community		Professionals		Public Authorities		Others	
	*n*	Description	*n*	Description	*n*	Description	*n*	Description
**In-depth semi-structured interviews**	3	Roma project participants	3	representatives of USS Kosice ^1^	3	officers from the City Council of Kosice	1	priest at Kosice-Lunik IX ^2^
	-	-	-	-	1	local authority of Velka Ida ^3^	1	cultural anthropologist
**Informal unstructured interviews**	2	wives of project participants	-	-	-	-	1	nun at Kosice-Lunik IX
	3	inhabitants from the segregated settlement in Velka Ida not participating in project	-	-	-	-	-	-
	2	community workers in Velka Ida	-	-	-	-	-	-
**Focus groups**	17	Roma children from the elementary school in Velka Ida	5	teachers at the elementary school in Velka Ida	6	representatives/workers at the Labour Office in Kosice	-	-
**Direct observation**	ca. 25	Roma job seekers during the recruitment process	3	representatives of USS Kosice during recruitment process	-	-	-	-
	ca. 50	inhabitants of Velka Ida	-	-	-	-	-	-
	ca. 20	inhabitants of Kosice-Lunik IX	-	-	-	-	-	-

Note: ^1^ U.S. Steel Kosice; ^2^ Kosice-Lunik IX is a city district of Kosice close to USS Kosice which is the largest Roma urban settlement in Slovakia; ^3^ Velka Ida is a village in the immediate vicinity of USS Kosice with a segregated Roma settlement.

**Table 2:** Variables and questions in the data collection based on the CMO framework, in particular regarding well-being and health as an outcome.

Variable	Questions
Outcomes	What do you think, what are the (positive and negative) outcomes of this project? What do you think, does the project have an impact on the rest of the community? If yes, why and how? If not, why?
Well-being and health	Do you think the project improves the chances of participants, their families and children to be healthier? If yes, why and how? If not, why? Are the project participants in a better condition than those from their surroundings who are not in the project? If yes, why? If not, why? Do the project participants have better health and living conditions than those from their surroundings who are not participating in the project? If yes, why? If not, why?

**Table 3:** Characteristics of the project participants.

Characteristics *		Share (%)
Education	primary school	56%
	secondary school without graduation	18%
	secondary school with graduation	26%
Age structure	18–20	8%
	21–30	38%
	31–40	33%
	41–50	13%
	51–60	8%
Locality	Velka Ida ^1^	52%
	Kosice-Lunik IX ^2^	19%
	Kosice-Saca ^3^	29%

Note: * The average number of project participants per year was 111; ^1^ Velka Ida is a village in the immediate vicinity of USS Kosice with a segregated Roma settlement; ^2^ Kosice-Lunik IX is a city district of Kosice close to USS Kosice, and is the biggest Roma urban settlement in Slovakia; ^3^ Kosice-Saca is a city district of Kosice with a Roma urban ghetto close to USS Kosice.

**Table 4:** Examples of quotes illustrating findings regarding the increased employability and the improved health and well-being of segregated Roma as outcomes of a public–private partnership.

Quotes	Group of Outcomes
*“It is important to me, that my sons attend school regularly and learn well, because without school they will not find a job. Maybe I’m hard on them, but it is for their own good. You know, those who do not work are not so tough on their kids, but then they do not go to school.”* Project participant, in-depth semi-structured interview [outcome: education].	Increased employability of segregated Roma
*“My father works in the project and our family is therefore well, certainly better than those children whose fathers do not work. I’m proud of him. I’m also learning well, so I can then continue to study and also find a good job.”* Roma child from the elementary school in Velka Ida, focus group [outcome: education].	
*“We* [USS Kosice] *closely cooperate with local primary schools, not only by monitoring school attendance and the behaviour of project participants’ children, but also by involving all schoolchildren in various projects, attempting to motivate them to complete primary school education and continue their studies at least at partner vocational schools.”* Representative of USS Kosice, in-depth semi-structured interview [outcome: education].	
*“I can see how they* [non-Roma colleagues from core staff] *treat me* [equally]. *They count with me. They treat me as a core employee not as a temporary worker. Foreman* [coordinator] *has even more confidence in me than in others.”* Project participant, in-depth semi-structured interview [outcome: social inclusion; increasing of self-confidence and courage].	
*“The fact that they* [segregated Roma] *come to the selection procedure and make contact with the mayor or his deputy and the other recruiters is already a first step toward making them feel more confident. Many of them, even the unsuccessful candidates, then often later seek out these people and ask their advice in various areas.”* Representative of USS Kosice, in-depth semi-structured interview [outcome: social inclusion; increasing of self-confidence and courage].	
*“Aside from finding work, they* [project participants] *come into contact with adults who are outside their community, who may have information, options, can offer encouragement, provide support, assistance with various things—because in their community they often cannot find an ‘expert’ for solving various problems.”* Local authority of Velka Ida, in-depth semi-structured interview [outcome: social inclusion; increasing of self-confidence and courage].	
*“Yes, it has been better since I worked at USS Kosice. I have regular income* […], *it is better in every way* […], *I have motivation there. I want to join the core staff once in the future. I see they have better salaries there; you know [smile]. So that is the better motivation there, you know* [smile]. *But I do not complain, it is good, still better than material needs’ 60 Euros* [material needs benefit payment provided by the Office of Labour, Social Affairs and Family for unemployed or low-income families]. Project participant, in-depth semi-structured interview [outcome: stable income].	Resulting better well-being and health of segregated Roma
*“The project ensured a stable income for many people. They* [project participants] *can now afford to buy many more things, such as clothes and school materials and tools for children, the absence of which was often previously a barrier to their school attendance.”* Community worker, informal unstructured interview [outcome: stable income].	
*“Certainly their* [project participant´s] *quality of life has improved through income. They have more income; they can afford more.”* Priest at Lunik X, in-depth semi-structured interview [outcome: stable income].	
*“The project is profitable. They* [project participants] *have a stable income and the collective agreement of the USS Kosice also applies to project participants, so they have 13th and 14th months´ salaries and rewards.”* Local authority of Velka Ida, in-depth semi-structured interview [outcome: stable income].	

## Appendix A. Stages of the Performed Qualitative Study

**Phase**	**Description**		**Time-Table**
1. Study protocol	elaboration of study protocol: objectives of the study, timetable set-up, field collection procedures, data collection procedures, analytic strategy		January 2013
2. Preparation for data collection	specifying of sites to be visited, data collection plan, contacting the respondents		February–March 2013
3. Data collection	collecting and studying of internal documents and previous studies (relevant academic and grey literature)		March–September 2013 January–October 2015 June–December 2016 January 2017–February 2018 March 2018–May 2019
	direct observation	presence in recruitment process	March 2013
		visit to settlements (Velka Ida, Lunik IX)	April; June; November 2013 January; September 2014 December 2017 March 2018
	in-depth face-to-face semi-structured interviews		April–July 2013
	focus groups		April–July 2013
	face-to-face unstructured interviews		November 2013
4. Data processing/analysis	content analysis: transcription, coding, recursive abstraction (searching for themes, reviewing themes, defining and naming themes), writing and finalising		July 2013–January 2014 June–October 2015 June–December 2016 January 2017–February 2018 March 2018–May 2019

## Appendix B. Structure of the Semi-Structured Interviews

1. Project initiation—human resources, financial assurance, social situation, legislation, material and technical support, locality…

(a) What are the circumstances of the project initiation? Why do you think this project was created? (physical neighbourhood, high crime, high unemployment rate, available and cheap labour force…) [*Why and how was the project initiated?*]
(b) Which factors, elements and mechanisms (tools, instruments) affected the project start-up in a positive and a negative way? (legislation, local authorities, financial assurance, material and technical support…) [*Why and how has the project initiation been supported? Why and how has the project initiation been limited and restricted?*]

2. Project implementation and maintenance—human resources, financial assurance, social situation, legislation, material and technical support, locality…

(a) Which factors, elements and mechanisms (tools, instruments) have enabled project implementation and maintenance (sustainability)? [*Why and how was the project implemented? Why and how has the project been maintained?*]
(b) Which factors, elements and mechanisms (tools, instruments) have precluded and hindered project implementation and maintenance? (Legislative—existing social support scheme, Labour Code, fluctuation…) [*Why and how has project implementation and maintenance been supported? Why and how has project implementation and maintenance been limited and restricted?*]

3. Project impact and outcomes

(a) What do you think, what are the (positive and negative) outcomes of this project?
(b) What do you think, does the project have an impact on the rest of the community? If yes, why and how? If not, why?

4. Well-being and health (health equity)

[*Why and how has the project affected health (health equity)? How may the project affect health (health equity)?*]

(a) Do you think the project improves the chances of participants, their families and children to be healthier? If yes, why and how? If not, why?
(b) Are the project participants in better shape than those from their surroundings who are not in the project? (Those employed and unemployed who are treated only by existing health-care system etc.). If yes, why? If not, why?
(c) Do the project participants have better health and living conditions than those from their surroundings who are not participating in the project? (Those employed and unemployed who are treated only by existing health care system etc.) If yes, why? If not, why?

**Specific investigator´s questions to keep in mind during the data collection!!!!**

*What is the context of the project initiation? What is the context and what are the elements and mechanisms that enabled the successful implementation of the project and its maintenance?*

*What is the context and what are the elements and mechanisms that precluded and hindered the successful implementation of the project*? (Barriers and limits of project initiation, implementation and maintenance).

What is the connection between the project and health equity? (Does the project have the impact on health equity?)

## Appendix C. Scenario of the Focus Group Interviews

1. Presentation of the researchers,

2. Presentation of the study focus,

3. Short presentation of the participants

4. Questions:

(a) Project creation
  - Why do you think this project was created? (What are the circumstances of the project initiation?)
  - Do you know how this project was created?
  - Were there any obstacles at the beginning? (Which factors, elements and mechanisms affected the project start-up in a negative way?)
  - Was there any support at the beginning? (Which factors, elements and mechanisms affected the project start-up in a positive way?)
(b) Project now
  - Are there any obstacles now? (Which factors, elements and mechanisms preclude and hinder the project maintenance?)
  - Who or what presents the main support of the project? (Which factors, elements and mechanisms enable and support the project maintenance?)
(c) Project outcomes
  - What do you think, what are the (positive and negative) outcomes of this project? (What are the positives about this project? What are the negatives about this project?)
  - What do you think, does the project have an impact on the rest of the community? If yes, why? If not, why?
(d) Project versus well-being and health
  - Do you think the project improves the chances of participants, their families and children to be healthier? If yes, why and how? If not, why?
  - Are the project participants in better shape than those from their surroundings who are not in project? (Those employed and unemployed who are treated only by the existing health care system etc.). If yes, why? If not, why?
  - Do the project participants have better health and living conditions than those from their surroundings who are not in the project? (Those employed and unemployed who are treated only by existing health-care system etc.) If yes, why? If not, why?
